# Hierarchical Microstructured K_3_V_2_(PO_4_)_3_/C‐Composite Electrode for Potassium‐Ion Batteries through Scalable Spray‐Drying Approach

**DOI:** 10.1002/cssc.202501111

**Published:** 2025-07-12

**Authors:** Andreas Heyn, Celine Röder, Holger Geßwein, Ali Ahmadian, Martin Velazquez‐Rizo, Nicole Bohn, Fabian Jeschull, Joachim R. Binder

**Affiliations:** ^1^ Institute for Applied Materials Karlsruhe Institute of Technology Herrmann‐von‐Helmholtz‐Platz 1 76344 Eggenstein‐Leopoldshafen Germany; ^2^ Helmholtz Institute Ulm for Electrochemical Energy Storage Karlsruhe Institute of Technology 89081 Ulm Germany; ^3^ Karlsruhe Nano Micro Facility Karlsruhe Institute of Technology 76344 Karlsruhe Germany; ^4^ Institute of Nanotechnology Karlsruhe Institute of Technology Herrmann‐von‐Helmholtz‐Platz 1 76344 Eggenstein‐Leopoldshafen Germany

**Keywords:** cathode materials, composites, hierarchical structures, microstructures, potassium‐ion‐batteries, potassium‐vanadium‐phosphates

## Abstract

Potassium vanadium phosphate K_3_V_2_(PO_4_)_3_ (KVP) is a promising polyanionic cathodic material for potassium‐ion batteries. As many other polyanionic materials KVP suffers from low electronic conductivity and shows just limited electrochemical performance. To overcome this limitation and improve the electrochemical performance, an easily scalable spray‐drying process is developed to create hierarchically structured KVP/C composites. The spray‐drying process leads to spherical and open porous granules of KVP particles wrapped in a carbon matrix, formed by the decomposition of sucrose and *β*‐lactose. The influence of different carbon sources (sucrose and *β*‐lactose) onto the granules’ microstructure is systematically studied and correlated it with the electrochemical performance of the KVP/C. The best‐performing composition is employed to study different electrolyte additives with the aim to improve the electrolyte stability at high potentials in potassium half‐cells.

## Graphical abstract

By systematically varying the carbon source contents the microstructure and morphology of the granules can be adjusted leading to optimized electrochemical performance.

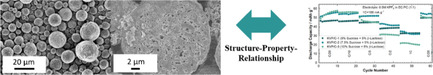

## 1 Introduction

Potassium ion batteries (PIBs) are a rapidly emerging type of alkali‐metal‐ion batteries.^[^ ^1^ ^]^ Beside their higher possible working voltage due to the lower alkali‐metal potential,^[^ ^2^ ^]^ the high abundance of potassium,^[^ ^3^ ^]^ the small ionic radii of solvated ions in the electrolyte (improved fast charge)^[^ ^4^ ^]^ and the utilization of graphite as an anodic material makes them an ideal candidate to complement lithium‐ion‐technology.^[^ ^5^ ^]^ Several anode materials have been studied extensively and showed stable cycling performance.^[^ ^5^, ^6^ ^]^ The development of high‐performance PIBs is currently still limited by a lack of suitable stable cathodic materials with scalable synthesis processes.^[^ ^7^ ^]^ Because of the much larger ionic radii of K^+^ (1.38 Å) compared to Na^+^ (1.02 Å) and Li^+^ (0.78 Å),^[^ ^4^ ^]^ a stable crystal structure/3D open framework and large K^+^ diffusion channels are necessary for a fast K^+^ migration and stable cycling performance, which seems to be the bottleneck for PIBs.^[^ ^7^, ^8^ ^]^ Besides Prussian blue analogs,^[^ ^9^ ^]^ transition metal oxides^[^ ^10^ ^]^ and organic compounds,^[^ ^11^ ^]^ polyanionic materials are another material group for the use as a PIB cathode material. One of the most promising polyanionic materials are vanadium‐phosphates of the chemical formula A_3_V_2_(PO_4_)_3_ (A = Li, Na, K). They have been explored as a cathodic material for LIBs and SIBs batteries successfully in the past decades and gained recent attention as a possible cathodic material for PIBs as well.^[^ ^12^ ^]^ Vanadium‐based phosphatic materials offer multiple valence states (+III ‐ +V), high working potential, and thermal stability due to the inductive effect of the PO_4_‐groups, but lack in electronic conductivity, resulting in poor C‐rate capability and capacity retention.[^7^, ^12^, ^13^]

To address these issues, different approaches have been applied. The most common ones are creating micro‐ and nanostructures or to cover the crystalline particles in a carbon coating.[^7^, ^12^] K_3_V_2_(PO_4_)_3_/C‐composites were first synthesized as nanowires and tested in SIBs by Wang et al. These nanostructures delivered an initial capacity of 119 mAh g^−1^ in SIBs and retained 99% of their capacity after 2000 cycles, but the crystal structure of the material remained unclear.^[^ ^14^ ^]^ In 2017, Han et al. used K_3_V_2_(PO_4_)_3_/C‐composites as a cathode in PIBs and studied the impact of carbon‐coated nanostructures created by freeze drying onto the electrochemical performance. The composites delivered an initial discharge capacity of 54 mAh g^−1^, while still 52 mAh g^−1^ remained after 100 cycles. In the case of KVP, there is still an ongoing discussion about the crystal structure of the compound.^[^ ^15^ ^]^ For instance, Zhang et al. proposed the crystal structure of KVP to be similar to the $R\bar{3}\text{C }$structure of the NASICON material Na_3_V_2_(PO_4_)_3_ by the use of in situ synchrotron X‐ray diffraction (XRD) analysis. Further information, like atomic positions that are needed for an unambiguous structural analysis, were missing in this study.^[^ ^16^ ^]^ In contrast, Jenkins et al. suggested a Pnna‐type orthorhombic crystal structure for the composites, assuming that all reported K_3_V_2_(PO_4_)_3_/C‐composites so far consist of K_3_V_3_(PO_4_)_4_*H_2_O together with impurities like KVP_2_O_7_ or K_3_V(PO_4_)_2_.^[^ ^17^ ^]^ Based on the Pnna‐type orthorhombic crystal structure, Kuai et al. studied the influence of calcination temperature in a citric‐assisted solgel synthesis onto the electrochemical performance of nanoporous K_3_V_2_(PO_4_)_3_/C‐composites. The materials calcined at 800 °C showed an initial discharge capacity of 76 mAh g^−1^ and good rate performance with 47 mAh g^−1^ of discharge capacity at 200 mA g^−1^.^[^ ^18^ ^]^ However, the influence of different carbon contents onto microstructure of the K_3_V_2_(PO_4_)_3_/C‐composites and the electrochemical performance was not methodically studied so far.

Aim of this paper is to develop an easily scalable solid‐state synthesis process of hierarchically structured KVP/C composites as a polyanionic cathode materials for PIBs. Therefore, we systematically studied the influence of carbon source content onto microstructure at different steps in the process and correlated the microstructural properties with the electrochemical performance. Furthermore, we investigated the phase evolution of KVP/C by combined thermal and fourier‐transform infrared spectroscopy (FT‐IR) analysis. XRD and transmission electron microscopy (TEM) analysis were performed to study the crystallinity of the KVP/C as a cathode material for PIBs.

## 2 Results and Discussion

**Figure 1:**
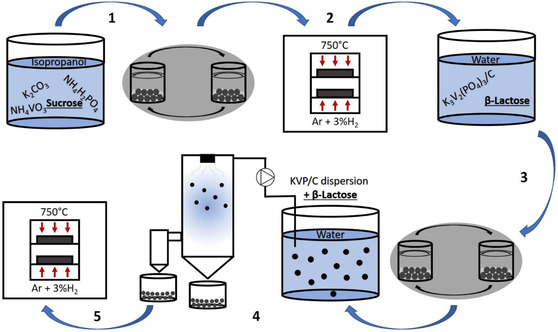
Schematic drawing of the synthesis process of hierarchically structured KVP/C‐composites with: 1) mixing of reactants by planetary ball milling in isopropanol with subsequent drying, 2) calcination under Ar + 3% H_2_‐atmosphere in a chamber oven at 750 °C, 3) mixing and grinding of KVP/C with *β*‐lactose in water by planetary ball milling, 4) spray‐drying of the KVP/C dispersion in water with *β*‐lactose, and 5) sintering of hierarchical structured secondary KVP/C particles under Ar + 3% H_2_‐atmosphere in a chamber oven at 750 °C.

The KVP/C‐samples were all synthesized via a carbothermal reduction process. To achieve an open porous microstructure of the carbon composites and improve the electrochemical performance, a spray‐drying process with subsequent thermal treatment (sintering) was applied. The synthesis process is schematically shown in **Figure** **1**, where in process step 1 and 3 the carbon source content was varied.

### 2.1 Synthesis of Hierarchically Structured KVP/C Composites

#### 2.1.1 Thermal and Structural Characterization of the KVP/C Phase

**Figure 2:**
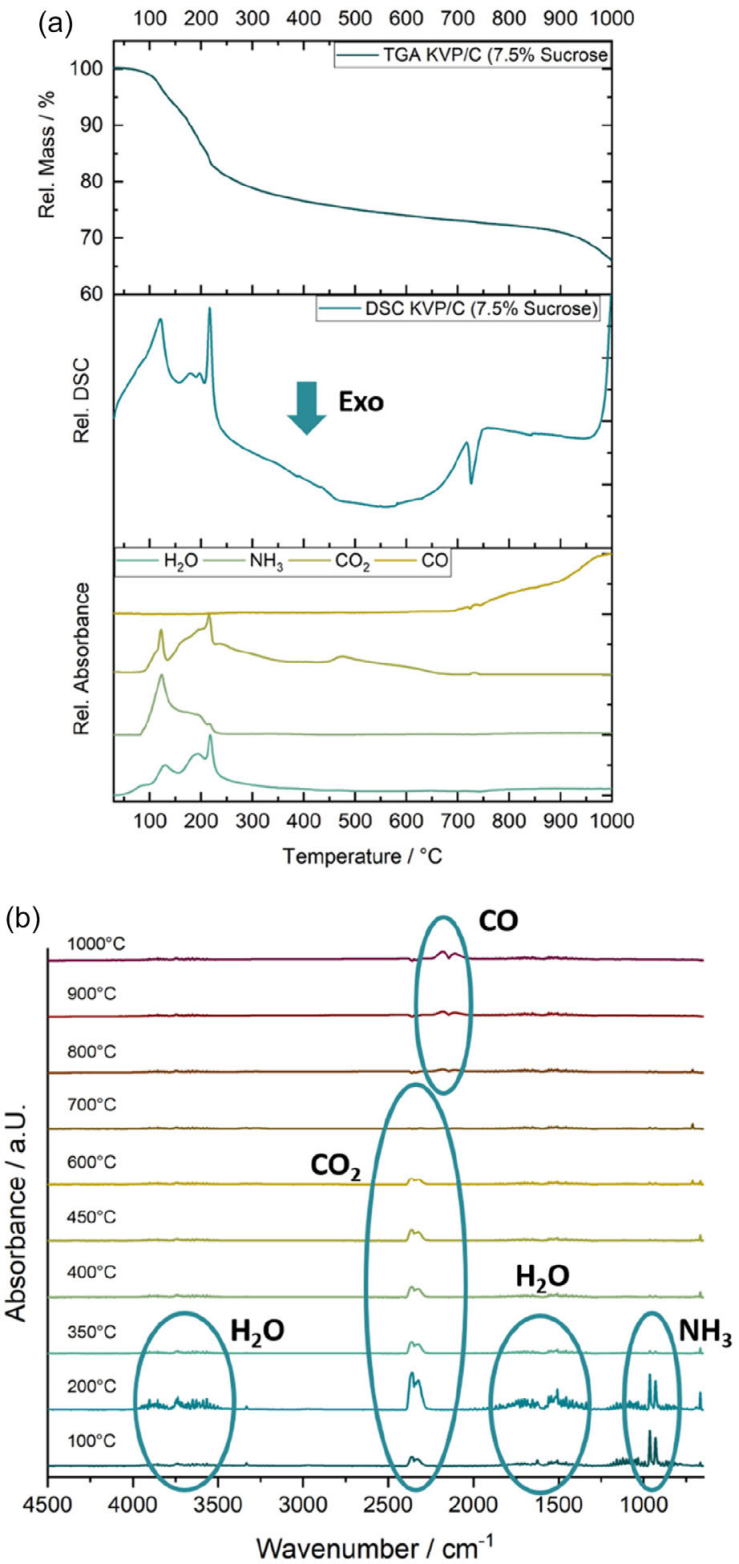
TG/DSC‐IR analysis on KVP/C‐2 precursors under Ar + 3 % H_2_ atmosphere. a) TGA (upper), DSC (middle), and FT‐IR‐traces of main gaseous byproducts (lower) and b) FT‐IR‐spectra with marked gaseous byproducts at different temperatures during thermal analysis.

The TGA/DSC coupled with FT‐IR results are shown in **Figure** **2**. It clearly shows the release of NH_3_ and H_2_O until 350 °C, followed by CO_2_ emission until 600–700 °C. This indicates the decomposition of the reactants NH_4_H_2_PO_4_, NH_4_VO_3_, and K_2_CO_3_, which correlates well with literature. At ≈800 °C, a continuous loss of CO rather than CO_2_ is observed, suggesting a possible reaction between KVP and the carbon coating leading to impurity phase formation.[^12^, ^19^] Additionally, an exothermic peak in the differential thermal analysis becomes visible around 700 °C (Figure 2). This indicates the beginning of the crystallization of the KVP‐phase. XRDs of KVP/C‐samples calcined at lower temperatures show no visible reflections, indicating an amorphous phase below 700 °C (Figure S1, Supporting Information) and confirming the thermal analysis results. As a consequence, for calcination of the KVP/C‐samples, a temperature of 750 °C was chosen. This temperature is between the crystallization at around 700 °C and further decomposition at temperatures above 800 °C of the KVP/C phase.

Theoretically, in the absence of added carbon sources, a reaction turnover of around 64% is expected. After the TG/DSC analysis, a residual relative mass of 66% is left, which correlates well with the theoretical reaction turnover. The difference between the values could arise from the residual carbon matrix from the carbonized sucrose. If a complete carbonization of the sucrose is assumed, a residual carbon content of 3.2% is expected. For example, the carbon content of the same KVP/C as used for the thermal analysis with 7.5% sucrose after 5 h calcination at 750 °C under Ar + 3% H_2_ atmosphere was 2.3% (Table 2). Assuming the reduction of vanadium is only caused by carbon, according to Equation (1) 1.5% of carbon is necessary for the complete reduction of V(+V) to V(+III) in the KVP. This leads to a theoretical carbon content after calcination of 1.7% for KVP/C‐2 & KVP/C‐5. Differences from the theoretical carbon content could arise due to a partly reduction of the vanadium by the reductive Ar + 3% H_2_‐atmosphere. The amount of carbon used for the reduction of vanadium decreases from 1.2% (KVP/C‐1 & KVP/C‐4) to 0.4% (KVP/C‐3 & KVP/C‐6) (Table S1, Supporting Information). This could be explained by higher specific surfaces of the carbon composites after calcination leading to a higher reactive surface and increased reduction of the vanadium by the reductive Ar + 3% H_2_‐atmosphere. **Table** **1** summarizes the weight loss, onset temperatures, and associated decomposition products.

**Table 1:** Weight losses and onset temperatures determined by thermal analysis combined with decomposition products.

Onset temperature [°C]	Weight loss [%]	Decomposition product/reaction process
100	21.1	NH_3_, H_2_O, CO_2_
300	6.6	CO_2_
700	–	Crystallization KVP
800	6.3	CO, possible reaction of carbon with KVP

Based on the results of thermal analysis the following reaction scheme is proposed Equation (1):
(1)

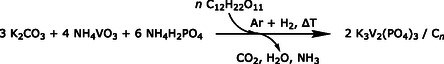




**Figure 3:**
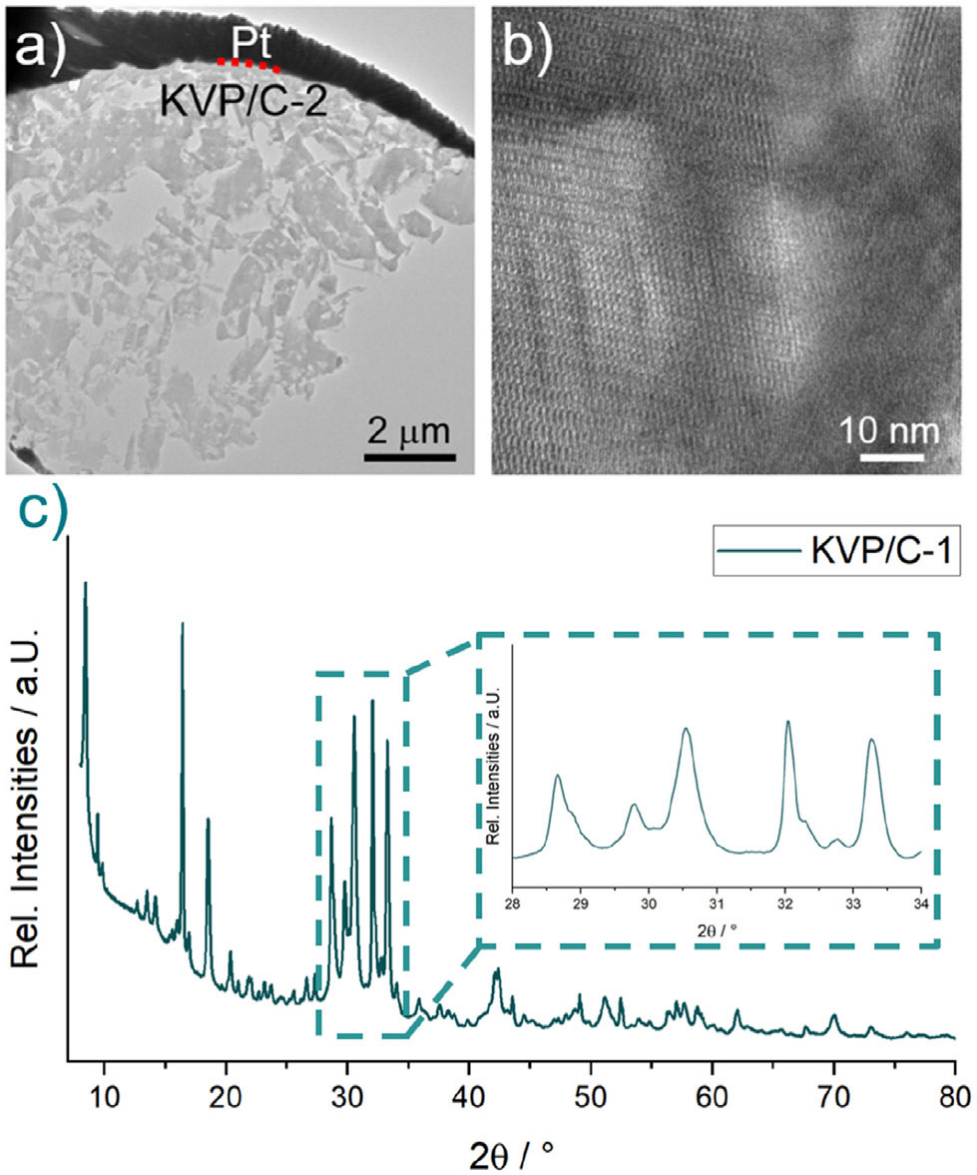
a) Prepared TEM‐lamella of a typical KVP/C‐2 granule, b) HR‐TEM image of the lattice fringes of KVP/C‐2, and c) collected XRD pattern of KVP/C‐1.

The powder diffraction pattern of the calcined KVP/C‐sample is shown in **Figure** **3**c. The diffraction peaks are relatively broad, which indicates small crystallite sizes and the presence of microstrains in the material. The small crystallite sizes are confirmed by TEM images presented in Figure 3a,b, together with the imaging of the lattice fringes by HR‐TEM. It should be noted that due to the porosity of the material (which limited the thinning‐down process during lamella preparation) and the small grain size of the crystallites, it was not possible to obtain direct single‐crystalline low‐index TEM imaging. An additional ICP‐OES analysis confirms the stoichiometry of the samples with a K:V:P‐ratio of 3:2:3 (Table S3, Supporting Information).

A visual comparison with the diffraction pattern of a K_3_V_2_(PO_4_)_3_/C composite synthesized by Zheng et al. shows a good match, especially in the enlarged view of the angular range between 28° and 34° 2θ.^[^ ^20^ ^]^ However, no crystallographic information is given in that study. Preliminary attempts to fit different structural models reported in the literature for K_3_V_2_(PO_4_)_3_ did not yield satisfying results, so the detailed crystallographic structure of our KVP/C composite remains unclear. We assume that our synthesis process is very sensitive to the used calcination atmosphere. Together with the presence of a carbon source, small changes in the atmosphere and reaction temperature can lead to an alteration of the vanadium valence state, which may change the crystallographic structure or may lead to the formation of different impurity phases. These issues will be addressed in ongoing investigations by our group.

In literature a discussion about the crystal structure of KVP is still ongoing. As one possible solution, the coexistence of at least two main phases of K_3_V_3_(PO_4_)_4_*H_2_O and K_3_V(PO_4_)_2_ instead of a phase in the stoichiometry of K_3_V_2_(PO_4_)_3_ is discussed. Depending on the calcination atmosphere and the stoichiometric ratios used for the synthesis, different main phases and impurities are found.^[^ ^17^ ^]^ However, some of the studies reveal some methodically weaknesses, as, for example, missing refinement parameters^[^ ^16^ ^]^ or the identification of crystal water inside the crystal structure.^[^ ^17^ ^]^

A more detailed structure solution for the highly porous KVP/C‐samples in this study remains challenging.

#### 2.1.2 Impact of Carbon Source Content onto Microstructure of KVP/C Composites

All samples were calcined and sintered at 750 °C under Ar + 3% H_2_ atmosphere and just the carbon source content was varied. The relevant powder characteristics of the different KVP/C‐samples are summarized in **Table** **2**.

**Table 2:** Summary of all relevant powder characteristics and amount of different carbon source used for the synthesis process of the KVP/C‐samples.

Sample	Sucrose [wt%]	*β*‐Lactose [wt%]	C‐content after Calcination [wt%]	C‐content after Sintering [wt%]	Median Secondary Particle Size [μm]	Specific Surface [m^2^/g]	Porosity [%]
KVP/C‐1	5	5	0.9	2.4	13.4	11.1	35.7
KVP/C‐2	7.5	5	2.3	3.6	13.6	20.7	38.3
KVP/C‐3	10	5	3.8	4.4	13.1	3.5	11.4
KVP/C‐4	5	15	0.9	4.4	14.9	41.1	30.4
KVP/C‐5	7.5	15	2.3	5.7	14.5	46.3	28.5
KVP/C‐6	10	15	3.8	7.2	13.9	56.3	30.9

**Figure 4:**
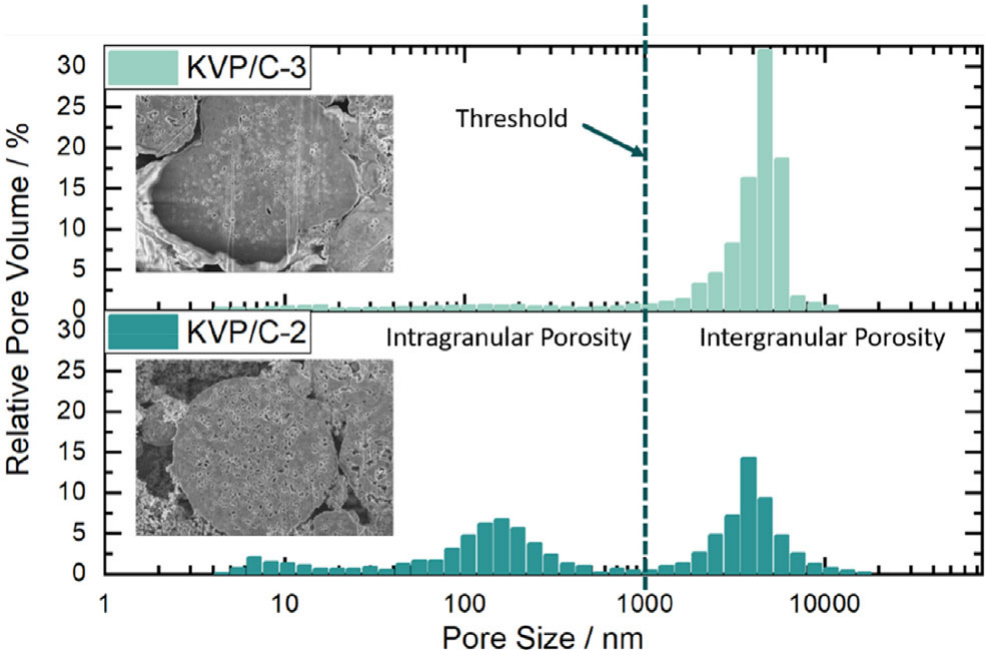
Pore Size Distribution of KVP/C‐2 and KVP/C‐3 with intra‐ and intergranular porosity. The threshold was used for the calculation of the total intragranular porosities.

**Figure 5:**
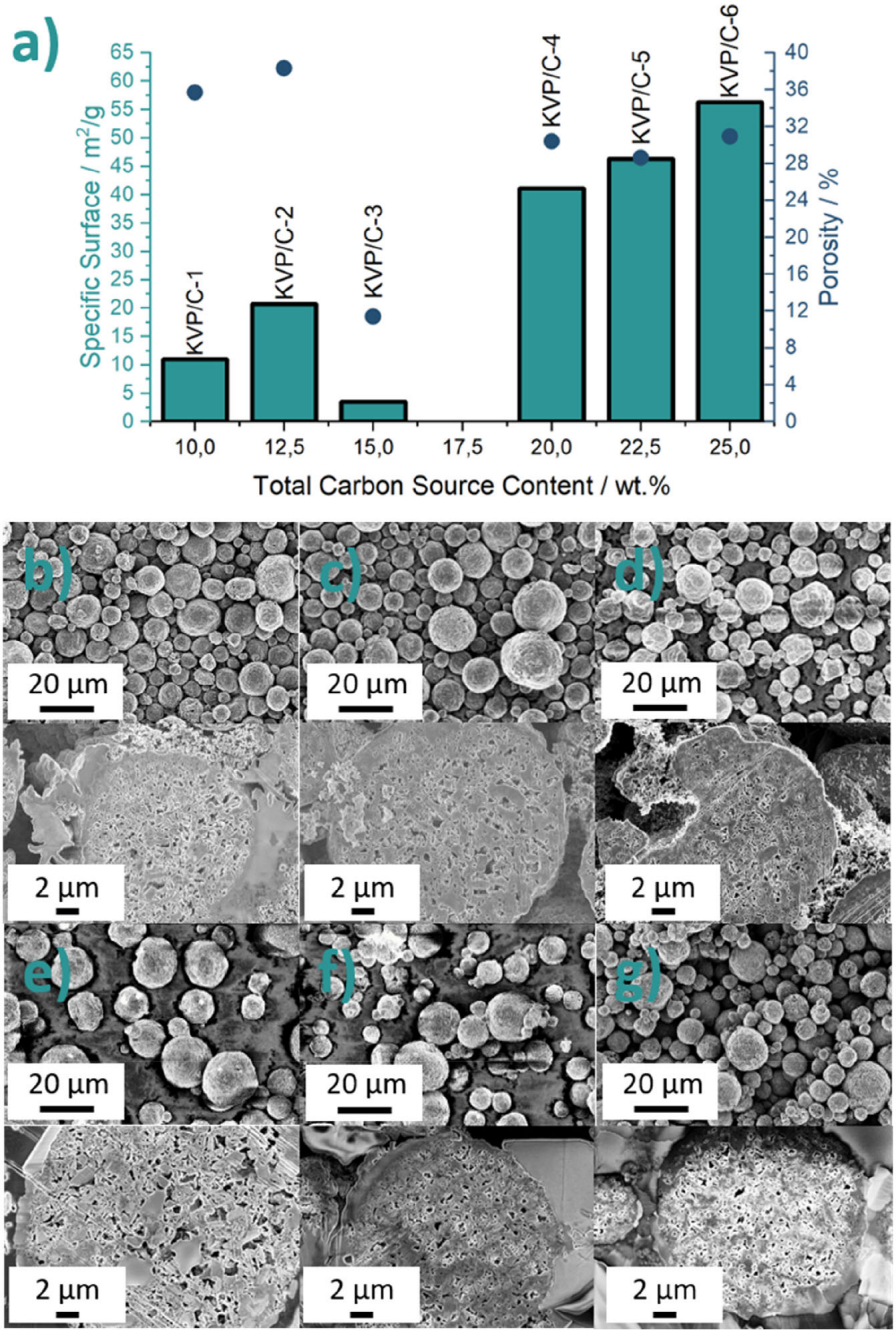
a) Correlation between specific surface (bars), porosity (bullets) and total carbon source content (sucrose + *β*‐lactose) after complete synthesis of all KVP/C‐composites and b–g) SEM images of the morphology and cross‐section of secondary particles with b) KVP/C‐1, c) KVP/C‐2, d) KVP/C‐3, e) KVP/C‐4, f) KVP/C‐5, and g) KVP/C‐6.

As expected, an increased total carbon source content leads to an increased carbon content after sintering, with values up to 7.2 wt% for sample KVP/C‐6 (Table 2). The median secondary particle size of the sintered granules seems to be nearly independent of the carbon source content, ranging from 13.1 to 13.9 μm for samples with 5 wt% of β‐lactose and from 13.9 μm to 14.9 μm for samples with 15 wt% of β‐lactose (Figure S2, Supporting Information). The increased carbon source content for the calcination leads to an increased specific surface of the KVP/C after calcination (Table S2, Supporting Information) and as well after completion of the whole synthesis process. This effect could be attributed to the decomposition and carbonization of the sucrose during the calcination process, which hinders particle growth and leads to nanosized crystallites of the KVP with high specific surface (Table S2 & Figure S3, Supporting Information).^[^ ^21^ ^]^ KVP/C‐3 seems to be an exception in terms of specific surface. The higher the amount of *β*‐lactose added before spray‐drying, the higher the specific surface after completion of the synthesis process. The addition of 15 wt% *β*‐lactose increases the specific surface up to 56.3 m^2^ g^−1^ for KVP/C‐6. But it should be noted that the stated specific surface is an effective area of the combination of carbon and KVP, so these composites are multiphase materials. Clear correlations between the stated specific surface and the electrochemical performance are not possible.^[12d]^ The porosity of KVP/C–1 and KVP/C‐2 with 35.7% and 38.3%, are higher than for the samples with 15% *β*‐lactose, with about 30% porosity (Figure 5a). KVP/C‐3 was found to exhibit an exceptionally low porosity (just 11.4%) and barely any open intragranular porosity (**Figure** **4**).

The porosity and morphology of the secondary particles are visualized by SEM images of cross sections and the particles itself (**Figure** **5**b–g). Inside the granules, an open porosity is visible for all samples, and the porosity is well distributed through the granules. For KVP/C‐3 seems to be an exception because no open porosity was visible. During spray‐drying of small primary particles, the smallest particles tend to arrange at the border and the surface of the granules. During the subsequent sintering process, these smaller particles sinter faster and form a dense area at the borders of the granules.^[^ ^22^ ^]^ This could be seen in the cross sections of all the samples, where a denser and less porous layer at the borders of the granules is visible. This was most clearly visible for sample KVP/C‐3, which showed a notably denser surface layer compared to the other samples (Figure S4, Supporting Information). This dense surface layer leads as a result to a much lower specific surface and porosity of sample KVP/C‐3 compared to all other KVP/C samples.

### 2.2 K‐Ion Storage Properties of KVP/C Composites

#### 2.2.1 C‐Rate Capability and Cycling Stability

**Figure 6:**
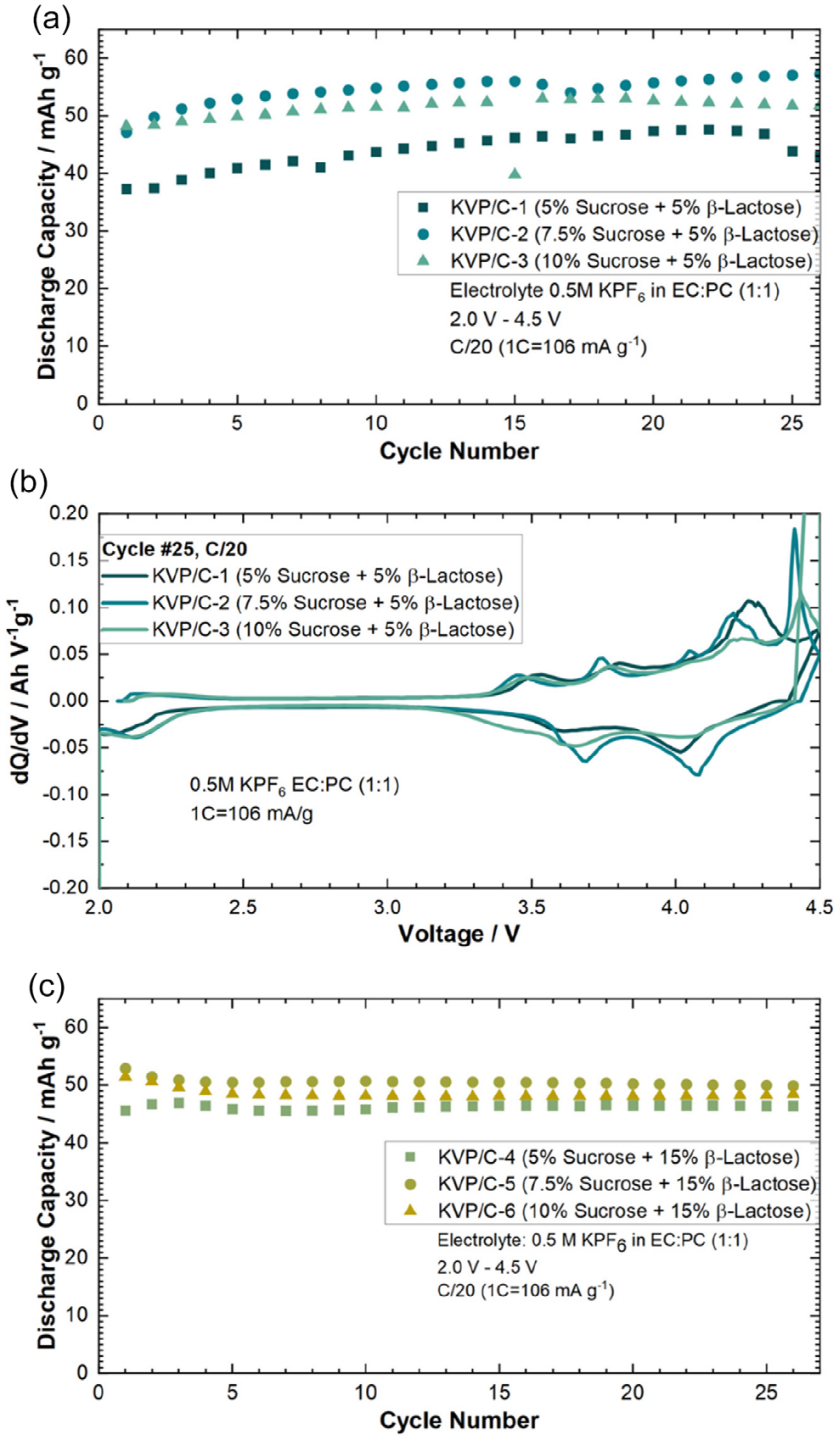
Comparison of specific discharge capacity of KVP/C depending on the sucrose‐content in PIB‐half cells for a) KVP/C‐samples with 5 % *β*‐lactose, b) dQ/dV‐Plot of KVP/C‐samples with 5% *β*‐lactose at Cycle 25, and c) discharge cpacity of KVP/C‐samples with 15 % *β*‐lactose.

The influence of different carbon source contents onto the electrochemistry of the KVP/C composites was evaluated in electrochemical tests in CR2032‐format potassium half cells in a voltage window of 2.0–4.5 V. Galvanostatic cycling was performed at a low C‐rate of C/20 (1C = 106 mA g^−1^) to determine the maximum discharge capacity, as shown in **Figure** **6**a,c. All stated capacities are referred to the KVP/C composite mass and not the KVP active material content. The corresponding Coulombic efficiencies are shown in Figure S5, Supporting Information. An increased sucrose content for the calcination step leads to an increased specific discharge capacity at C/20, while an optimum is observed with 7.5% sucrose. Composite KVP/C‐2 showed the highest specific discharge capacity with 58 mAh g^−1^ after 25 cycles, while the composites with only 5% sucrose, KVP/C‐1 (Figure 6a) and KVP/C‐4 (Figure 6c), showed the lowest discharge capacities. For all KVP/C composites with 5% *β*‐lactose, an increasing discharge capacity over 25 cycles is visible. This could be attributed to the activation of inactive particle domains. Compared to other studies on KVP/C, where the performance of primary particles was evaluated, our work deals with secondary granules, which are beneficial for further processing in large‐scale application. This could lead to some inactive domains at the beginning, which could be activated during continuous galvanostatic cycling.

For the KVP/C composites with 15% of *β*‐lactose (Figure 6c), the differences in the electrochemical performance are smaller than for the composites with 5% of *β*‐lactose. The total carbon content after sintering is probably above a critical value for samples with 15% of *β*‐lactose and overlays this effect partly.^[^ ^23^ ^]^ Therefore, an increase of the *β*‐lactose content to 15% in the spray‐drying and sintering process steps does not lead to any further increase in discharge capacity. Even if the discharge capacities are calculated without the carbon contents, the same trends remain visible (Figure S9, Supporting Information).

Rate capability tests were performed in the range from C/20 to 1C for the six KVP/C composites. The test was limited to rates of 1C for two reasons: Firstly, the highly reactive potassium metal counter electrode in half cells tends to build up high resistivity itself and is a main cause for high cell polarization in half cells. This causes an increasing bias in rate capability tests with increasing C‐rate.^[^ ^8^ ^]^ Secondly, the rate tests served as a means to identify viable C‐rates that do not suffer from rate‐induced capacity losses. Except for the KVP‐C3 composite, all compositions appear to sustain C‐rates up to C/5 without significant capacity losses.

While cycle life tests in PIB‐half cell setups are critical for a first material assessment, there are many practical problems associated with the use of potassium metal counter electrodes, including its high reactivity toward electrolyte components, rapid build‐up of resistive surface layers, as well as growth of dendrites and mossy structures. A particular problem is the poor passivation of the potassium metal electrode from recurrent electrolyte degradation processes that amplify the surface layer growth and also lead to side reactions from parasitic soluble species.^[^ ^24^ ^]^ Based on the microstructure of the KVP/C granules with its high specific surfaces, whereby more side reactions could take place, and the problems associated with potassium metal counter electrodes and surface layer formation from electrolyte degradation at high potentials, low initial coulombic efficiency (ICE) for all KVP/C compositions are visible (Figure S5, Supporting Information). Additionally, the voltage profiles on the first cycle (Figure S6, Supporting Information) could indicate that the material is oxidized in the best samples to a higher degree, but upon reduction a lower fraction of potassium intercalates back into the material (in other compositions, effects like the poorly accessible pore network further limits the capacity). This appears to be an intrinsic limitation of KVP and is observed in other studies as well.^[^ ^15^, ^18^ ^]^ Some electrochemically inactive domains at the beginning of the cycling could contribute to the low ICE as well, while they could get activated during continuous cycling as previously stated.

During cycle life evaluation, the CE of KVP/C‐2 at C/20 is approximately ≈95% for 100 cycles, indicating the problems associated with the potassium metal counter electrodes as previously stated. Nevertheless, based on this half‐cell results, KVP/C‐2 shows a good cycling stability at C/20 for 100 cycles, while 85% (51 mAh g^−1^) of the maximum discharge capacity remains in this half‐cell setup (Figure S7, Supporting Information).

**Figure 7:**
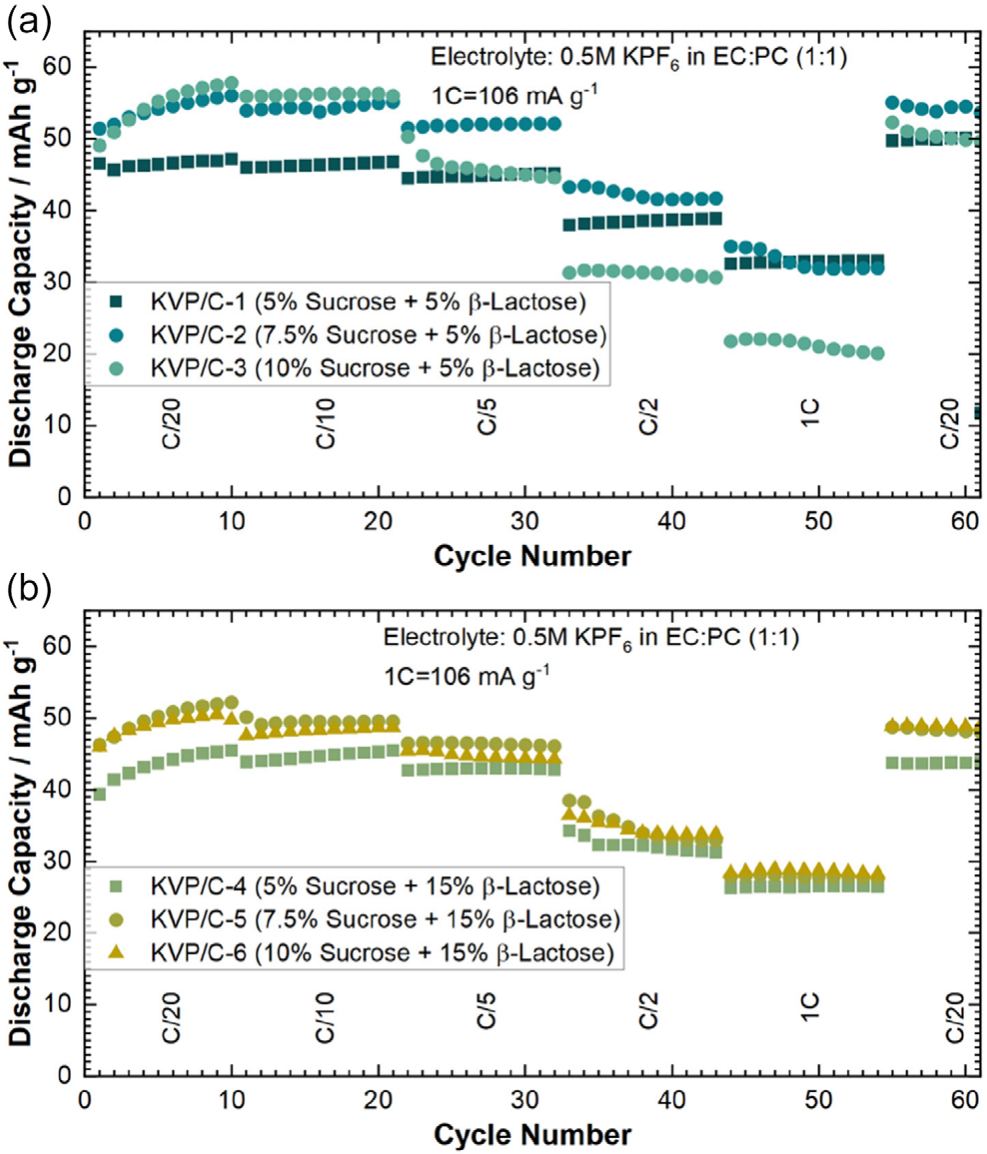
C‐Rate capability for all KVP/C‐samples depending on the carbon source content in PIB‐half cells with a) C‐rate performance for KVP/C‐samples with 5 wt% *β*‐lactose and b) C‐rate performance for KVP/C‐samples with 15 wt% *β*‐lactose.

**Figure 8:**
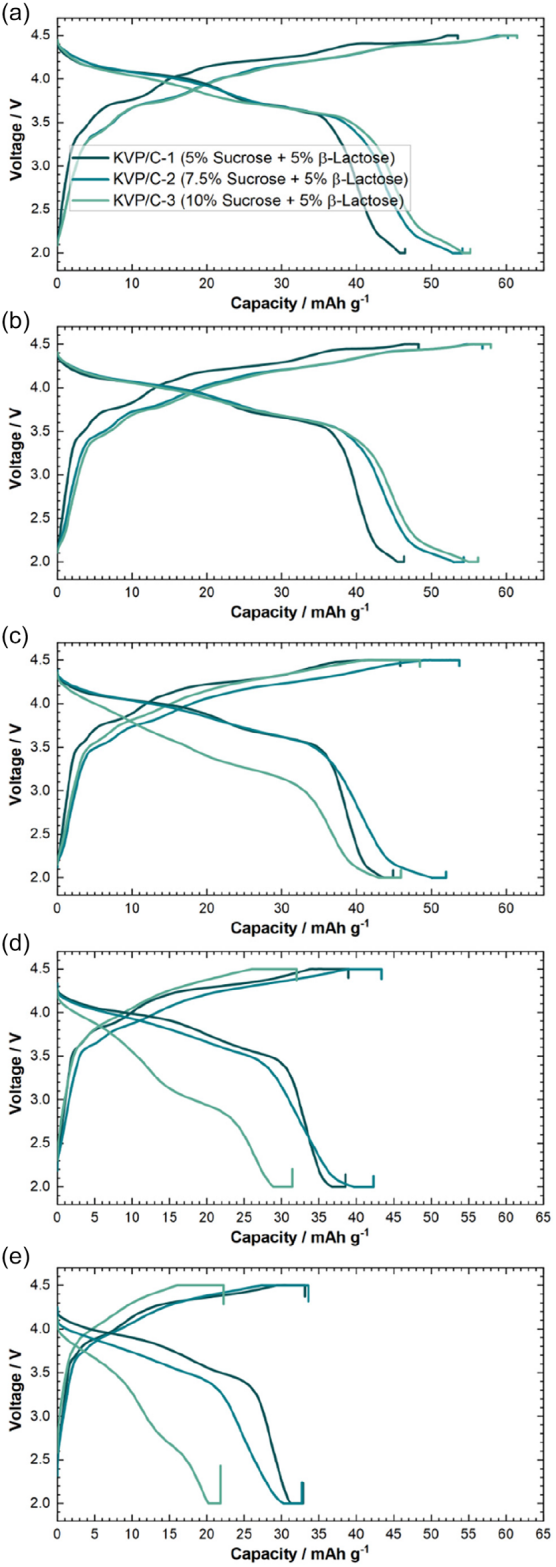
Voltage Profiles of KVP/C‐samples with 5 wt% of *β*‐lactose in PIB half cells at different C‐Rates in the 5th cycle at each C‐rate. a) C/20, b) C/10, c) C/5, d) C/2, and e) 1C. 1C = 106 mA g^−1^ and as an electrolyte 0.5 m KPF_6_ in EC:PC (1:1) was used.

KVP/C‐2 performs best at low and moderate C‐rates until C/2 with the highest discharge capacity (**Figure** **7**a). At 1C KVP/C‐2 and KVP/C‐1 showed a similar discharge capacity, while the C‐rate capability of KVP/C‐3 is the worst (Figure S7, Supporting Information). Not only displays KVP/C‐3 the lowest capacities, the voltage profiles undergo drastic changes at higher C‐rates due to polarization effects, as indicated in the comparisons in **Figure** **8**. The KVP/C‐samples with 15% of *β*‐lactose showed all a similar C‐rate capability, which seems to be an effect of better and nearly similar intragranular porosity. (Figure 7b). Compared to the corresponding KVP/C‐samples with 5% *β*‐lactose, the samples with 15% *β*‐lactose maintained generally lower discharge capacities at all C‐rates tested (Figure 7). Even if the different carbon contents of the KVP/C‐composites are considered and the discharge capacities of the samples are calculated without the carbon content, the same trends remain visible (Figure S10, Supporting Information). The corresponding dqdV‐plot and voltage profiles of the KVP/C‐samples with 15 wt% *β*‐lactose are shown in Figure S11 & 12, Supporting Information.

Summarizing the results above, it seems that a sucrose content of 7.5 wt% is the optimum, yielding a maximum specific discharge capacity of up to 58 mAh g^−1^ at C/20 after 25 cycles. A further increase in sucrose content does not lead to a further increase in discharge capacity. More importantly, an increase in sucrose content leads to a notably poorer performance in terms of C‐rate capability, maximum discharge capacity and polarization, which is likely linked to the microstructure of the KVP/C granules. For instance, KVP/C‐3 showing the poorest rate‐capability and likewise the lowest particle porosity. An increased sucrose content inhibits particle growth and leads to nano‐crystalline particles (Figure 3a,b).^[12d]^ Accordingly, an increase of the sucrose content above 7.5 wt% leads to thicker and denser carbon coatings at the surface of the primary particles. The thick carbon coatings could limit or inhibit K‐ion diffusion across the interface from the liquid electrolyte phase to the active material particle and thus render more domains of the KVP/C granules electrochemically inactive.[^23^, ^25^] This could be observed, for instance, in plots of the differential capacity dQ/dV for KVP/C‐1, KVP/C‐2 and KVP/C‐3 shown in Figure 6b, where a clear shift of the redox peaks during reduction to higher potentials are visible for KVP/C‐2 compared to KVP/C‐3 with less polarization. The higher intragranular porosity of 38.3% for KVP/C‐2 and 35.7% for KVP/C–1 can explain the improved C‐rate capability compared to KVP/C‐3 with just 11.4%. The thick carbon coating combined with the low intragranular porosity of KVP/C‐3 both contribute to poor mass‐transport properties in the electrolyte and particle phase and generate a poorly percolated electronic network in the particle matrix, which becomes apparent in significant polarization effects at higher C‐rates (Figure 8). Hence, the carbon coating of KVP/C‐2 in combination with the higher specific surface area and increased intragranular porosity, leads to a shortening of the diffusion length inside the KVP/C composites. This promotes electrolyte penetration and interfacial contact, which increases potassium‐ion diffusion and thus improves the depth of discharge, yielding higher specific discharge capacities at low C‐rates, as well as better C‐rate capability.[^12^, ^15^, ^18^, ^21^]

Our results suggest that KVP/C‐1 and KVP/C‐2 have an improved C‐rate performance compared to the samples with 15 wt% of *β*‐lactose added. This could be linked to the increased porosity of up to 38% for KVP/C‐2 leading to nanostructured granules and a shortening of the diffusion length inside the granules. The addition of 7.5 wt% sucrose and 5 wt% of *β*‐lactose is the optimum composition for creating hierarchically structured KVP/C composites for high‐voltage application in PIBs.

#### 2.2.2 Effect of Electrolyte

Previous works stated three major challenges in the material evaluation in half‐cell tests related to the reactive potassium metal negative electrode[^24^, ^26^] formation of soluble degradation products that initiate an internal shuttle process^[24a,d]^ and the electrolyte stability at high potentials.[^26^, ^27^] Specifically vanadium‐based positive electrodes (mostly KVPO_4_F) were also reported to suffer from notable degrees of vanadium dissolution.^[^ ^28^ ^]^ In the corresponding sodium‐ion systems, Mariyappan and coworkers^[^ ^29^ ^]^ suggested, amongst others, vinylene carbonate (VC) and tris(trimethylsilyl)phosphite (TMSP) as electrolyte additives to reduce the degree of irreversible reactions. In addition, electrolyte compositions of EC and PC, as were used in above experiments, appear to show generally higher cathodic and anodic stabilities compared to mixtures containing linear carbonates.[^26^, ^30^] For Prussian white materials, EC:PC mixtures with small amounts of fluoroethylene carbonate (FEC) have shown promising results.^[^ ^31^ ^]^ Taking these previous considerations into account, we performed an electrolyte additive screening on half‐cell level using the microstructured KVP/C active material prepared herein.

**Figure 9:**
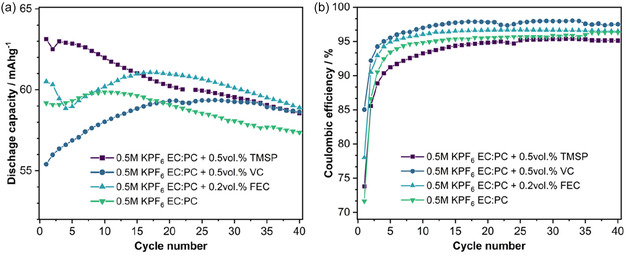
a) Discharge capacities and b) coulombic efficiency of the selected electrolytes containing various additives for sample KVP/C‐2.

Galvanostatic cycling experiments in K//KVP/C half‐cells were conducted to determine the impact of the above‐mentioned additives at a fixed cycling rate of C/20 within the potential window from 2.0 to 4.5 V (vs. K^+^/K). Multiple additive concentrations have been tested and are collected in Figure S13 & S14, Supporting Information, while discharge capacities and Coulombic efficiencies (CEs) of the best‐performing electrolyte compositions are presented in **Figure** **9**. The samples were tested against the EC:PC (v/v = 1:1) base electrolyte used in the previous sections that displayed an initial discharge capacity of 59 mAh g^−1^. Compared to the additive‐free electrolyte, other electrolyte compositions achieved only minor improvements of less than 2 mAh g^−1^ after 40 cycles. Their capacity decays converged into similar fading rates. The capacity scale in Figure 9 was enlarged (from 52 to 65 mAh g^−1^) to better visualize the differences. Interestingly, the electrolyte compositions with additives showed distinctly different cycling behaviors in the first 10–20 cycles, with TMSP providing the highest initial discharge capacity (63 mAh g^−1^) in this comparison. The VC‐containing electrolyte behaves the opposite, showing the lowest initial discharge capacity, followed by a gradual capacity increase over the next 20 cycles. This could point toward a total decomposition of the additives within these first 10–20 cycles and is likely linked to the high reactivity of the K negative electrode. The voltage profiles (Figure S15, Supporting Information) further suggest no significant build‐up of additional overpotentials, as the potential differences between the profiles lie within a margin of less than 10 mV. The overall lower capacity in VC‐ and FEC‐containing electrolytes are likely a result of reduced degrees of side reactions above 3.5 V, which shorten the length of the voltage profile features of KVP/C.

More significant differences are seen in the CEs of the investigated electrolyte formulations. The base electrolyte showed the lowest CE of 71.6% initially, which increased on the following cycles to up to 94.8%. Low CEs, that is, values considerably below 99% after a number of initial cycles, in half cell experiments are not uncommon and related to the high reactivity of potassium toward the electrolyte.[^27^, ^32^] The TMSP‐containing electrolyte reached similar low CEs after around 20 cycles. The electrolyte with VC additive displayed the highest initial CE, as well as the highest CEs (97.3%–98%) of all investigated electrolyte systems. Compared to the additive‐free and TMSP‐containing electrolyte, small amounts of FEC (0.2 vol%) also helped to improve the initial CE and to retain higher CEs over the investigated 40 cycles.

The results indicate that most additives in these half‐cell experiments work best at comparatively small concentrations of less than 1 vol%. The capacity retention for FEC becomes particularly poor when higher contents are used. While larger contents have been reported to inhibit the cell reaction entirely,^[^ ^33^ ^]^ it would appear that small amounts are rapidly consumed and yet provide a positive impact on the initial capacity and CE. A rapid consumption likely applies to the TMSP and VC additive as well. As known SEI‐forming additives VC and FEC will react at least partly at the K‐metal electrode. The resulting surface layers may slow down side reactions with other electrolyte components, as their polymeric decomposition products can contribute to the formation of a flexible and stable interface.^[^ ^34^ ^]^ This strongly suggests different natures of the formed interfacial layers. Especially the layer thicknesses and the individual layer components (e.g., organic/inorganic species) can greatly influence CE. TMSP could potentially benefit cells during conditioning by scavenging fluorine from PF_6_^−^ degradation reactions but may also induce side reactions with fluorine‐containing compounds as well.^[^ ^35^ ^]^ Any positive effect owing to its high oxidation potential^[^ ^36^ ^]^ and in supressing vanadium dissolution appears to dissipate within the first 10–15 cycles.

In summary, although FEC and VC are widely used in lithium‐ and sodium‐ion cell chemistries (half and full cells), they do not appear to have a long‐term effect on the capacity retention in potassium‐ion cells. On the contrary, larger concentrations of FEC are highly detrimental, thus highlighting challenges in implementing drop‐in solutions for potassium systems and the clear need for 3‐electrode studies to pinpoint the origins of parasitic processes.

## 3 Conclusion

In this work we successfully developed a synthesis process to design hierarchical structured KVP/C composites. A spray‐drying process with subsequent sintering leads to spherical KVP/C granules with open porosity. The phase evolution was studied by TG/DSC‐IR, while the crystallization of KVP starts at temperatures around 650 °C. A detailed structural analysis at this highly porous KVP/C‐samples remains challenging and subsequent investigations for precise structural information are planned. Further the influence of different amount of carbon sources onto the microstructure and the electrochemical performance was studied. The addition of 7.5 wt% sucrose and 5 wt% *β*‐lactose leads to an optimum composition of the KVP/C‐composites. This increases the open porosity of the granules to 38% with an increased specific surface as well, while it leads to the highest discharge capacity at C/20 (59 mAh g^−1^) and best C‐rate capability (KVP/C–2). Subsequently the addition of different electrolyte additives to a 0.5M KPF_6_ EC:PC electrolyte in potassium half‐cells with KVP/C was studied to stabilize the SEI at the K‐anode and prevent electrolyte degradation at high potentials. The addition of small amounts of TMSP, VC, or FEC lead to an increased CE and slightly higher discharge capacities at C/20.With this study we gained further insides into the crystallization of the KVP‐phase and developed an easily scalable synthesis process for the first time. This could be the starting point for further optimization of KVP for its use in PIB Full Cells in terms of optimizing the crystal structure or microstructure of the granules.

## 4 Experimental Section

4.1

4.1.1

##### Material Synthesis

As reactants K_2_CO_3_ (VWR, Germany), NH_4_VO_3_ (Thermo Scientific, USA) and NH_4_H_2_PO_4_ (VWR, Germany), as well as sucrose (Sigma–Aldrich, USA) and *β*‐lactose (Sigma–Aldrich, USA) as carbon sources, were used. First, the reactants K_2_CO_3_, NH_4_VO_3_, and NH_4_H_2_PO_4_ were thoroughly mixed in the stochiometric ratio of 3:4:6 together with different weight percentages of sucrose by ball milling in 2‐propanol in a planetary ball mill for 8 h. 300 g ZrO_2_ ball with a diameter of 3 mm were used for milling. Afterwards the 2‐propanol was evaporated and the mixture of reactants dried over night at 80 °C.

The calcination of the materials was performed under reducing Ar + 3% H_2_‐atmosphere in a retort oven. The heating rate was 1 K min^−1^ till 450 °C, where the temperature was held for 2 h. Afterwards the heating rate was increased to 3 K min^−1^ until 750 °C for the calcination of 5 h at this temperature. The samples were naturally cooled down to room temperature afterwards.

Subsequently the samples were prepared for the spray‐drying process to create hierarchical microstructures. Therefore, different amounts of *β*‐lactose were added to the composites, and the samples were ball milled in a planetary ball mill with deionized water as dispersion media. 300 g ZrO_2_ ball with a diameter of 3 mm were used for milling as well. For the following spray‐drying process, the solid content was adjusted to 8–10 wt%, and 0.5 wt% of PEG and PAA were added. The inlet temperature was 210 °C, the outlet temperature was 112 °C, as a process gas, nitrogen was used. The samples were spray‐dried with 30.000–35.000 rpm of the atomizer. The tower and cyclone fraction were combined and then subjected to a further heat treatment in a retort oven under reducing Ar + 3% H_2_‐atmosphere. The heating rate was 5 K min^−1^ till 750 °C, where the temperature was held for 5 h. Afterwards the samples were cooled naturally to room temperature, sieved, and stored in an exsiccator under vacuum.

##### Material Characterization

Thermal analysis was performed with a TGA STA449 (Netzsch, Germany) directly coupled with a FT‐IR Vertex 70 (Bruker, Germany) to analyze gaseous reaction products. The heating rate was 5 K min^−1^ till 900 °C under Ar + 3% H_2_‐atmosphere. The coupling and FT‐IR were heated to 200 °C both in advance.

Powder‐XRD measurements were recorded at a D8 quest (Bruker, Germany) by using Cu‐Kα‐rays between 10° and 80°.

The sample for TEM analysis was a lift‐out lamella prepared using a Strata 400 S (ThermoFisher Scientific) dual‐beam gallium focused ion‐beam (FIB)/SEM system. The surface of KVP/C‐2 granule was protected before preparation using electron‐ and Ga‐beam‐deposited platinum. The TEM measurements were conducted using a probe‐corrected Themis 300 TEM (ThermoFisher Scientific) operated at 300 kV in TEM mode. SEM images were recorded with a Supra55 SEM (Zeiss, Germany) at different acceleration voltages and magnifications.

All samples were predried under vacuum at 120 °C over night for evaluation of particle‐specific parameters. For determination of the specific surface of the particles N_2_‐sorption measurements at Gemini VII 2390a (Micromeritics, USA) were performed. BET‐evaluation was used in the linear region of the adsorption isotherm.

Mercuryporosimetry was used to measure the internal porosity of the particles with a CEI PASCAL 1.05 (Thermo Electron, USA). The internal porosity P was calculated by use of the internal specific pore volume V_P_ below a defined threshold value based on the following formula: $P=\frac{V_{p}}{V_{p}+\;\frac{1}{\rho }}$ with the density of the different composites *ρ*. The threshold was used to distinguish between intergranular and intragranular porosity.

The density of the composites ρ, was measured in preliminary experiments by the use of Mercury‐pycnometry with a Pycnomatic ATC (Thermo Fisher, USA).

Particle size distributions were measured by use of static light scattering with Horiba LA50 (Retsch Technology, Germany). Internal sonification was used to destroy possible agglomerates.

Chemical composition was evaluated by use of ICP‐OES with iCAP7600 duo (Thermo Scientific, USA). The carbon content was analyzed with a CS‐analyzer.

##### Electrochemical Characterization

For preparation of the working electrodes, the samples were first dried at 120 °C under vacuum in a vacuum oven (Buechi, Switzerland) and sieved with a 32 μm sieve. Subsequently, the KVP/C‐samples were mixed in a Speedmixer with a premixed slurry containing PVDF (Solef PVDF 5130/1001, Solvay), C65 (C‐Nergy Super C65, TIMCAL), and NMP in a ratio of KVP/C:C65:PVDF of 80:10:10. The well‐mixed and homogenous slurry was then casted onto Al‐foil with a wet‐film thickness of 200 μm by a doctor blade. The electrode sheets were cut into 12 mm disks and dried at 120 °C under vacuum in an Buechi oven. Mass loadings were between 3.5 mg cm^2^ and 4.5 mg cm^2^.

Coin‐cells of type 2032 in a half‐cell setup were assembled in Ar‐filled gloveboxes (H_2_O & O_2_< 0.1 ppm) subsequently. As working electrode, the different KVP/C‐samples were used, while potassium metal (Thermo Scientific, USA) was used as anode. The potassium metal was cut into small pieces, washed thoroughly in n‐hexane and stored over n‐hexane inside the glovebox. For preparation of potassium metal anodes, the potassium metal was rolled and squeezed into thin sheets inside a nitrile glove. After that, precut 14 mm disks of copper foil were stuck onto the potassium metal sheet, and 14 mm disk electrodes were punched out to use them as the anode.

As an electrolyte, a solution of 0.5M KPF_6_ in EC:PC (1:1 by volume) was used as the base, and TMSP (Sigma–Aldrich, ≥ 95%), VC (Thermo Scientific, 98%), and FEC (Sigma–Aldrich, > 99%, anhydrous) were evaluated as an additives. The electrolyte volume was set to 150 μl.

As a separator, Whatman GF/C‐separators with a diameter of 16 mm were used. Separators were predried in a Buechi oven at 120 °C under vacuum for 12 h.

Galvanostatic charging (CCCV) with a CV step was performed at a Biologic BCS (Biologic, France) in a voltage range from 2.0 to 4.5 V. The CV step was performed at the lower and upper cut‐off voltages of 2.0 V and 4.5 V, respectively. The cut‐off current for the CV step was defined as half the current that was applied at the respective C‐rate. The C‐rate was defined as 1C = 106 mA g^−1^ based on the theoretical capacity of the material. CCCV measurements were performed at the following C‐rates: C/20, C/10, C/5, C/2, and 1C for 10 cycles at each C‐rate. All stated capacities were referred to the KVP/C composite mass and not the KVP active material content.

## Conflict of Interest

The authors declare no conflict of interest.

## Supporting information

Supplementary Material

## Data Availability

The data that support the findings of this study are available from the corresponding author upon reasonable request.
