# Supplementary material for: Hierarchical Microstructured K3V2(PO4)3/C‐Composite Electrode for Potassium‐Ion Batteries through Scalable Spray‐Drying Approach
Source: ChemSusChem. 2025 Jul 12;18(16):e202501111. doi: 10.1002/cssc.202501111 (PMC12330327; doi:10.1002/cssc.202501111)
Supplement: Supplementary file 1 — Supplementary Material [file CSSC-18-e202501111-s001.pdf]

Supporting Information

Hierarchical Microstructured $K_3V_2(PO_4)_3$/C-Composite Electrode for Potassium-Ion-Batteries Through Scalable Spray-Drying Approach

Andreas Heyn, Celine Röder, Holger Geßwein, Ali Ahmadian, Martin Velazquez-Rizo, Nicole Bohn, Fabian Jeschull, Joachim R. Binder*

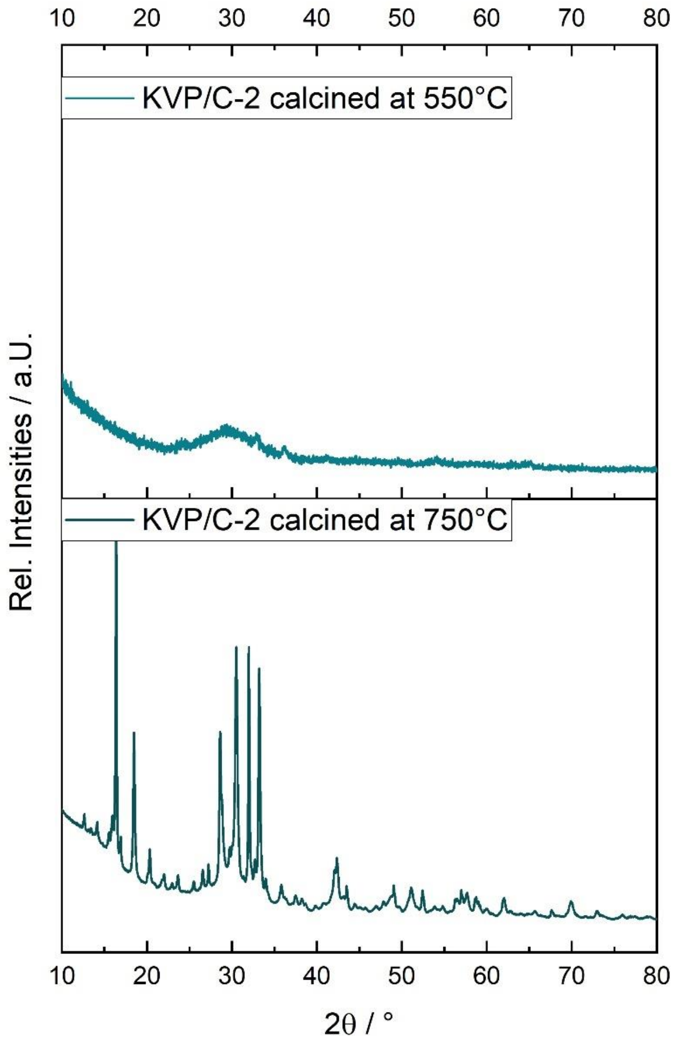

Figure S1: PXRD of KVP/C-2 precursors calcined at temperatures below 700 °C for 48 h under Ar + 3 % H_2 atmosphere.

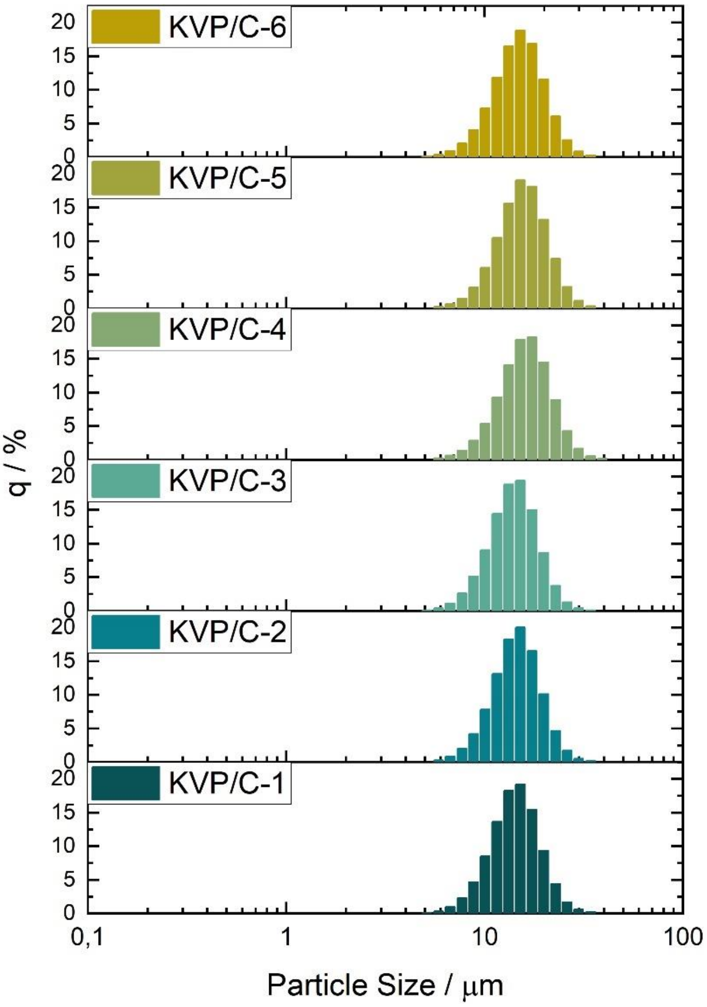

Figure S2: Particle Size Distribution for all different KVP/C-composites.

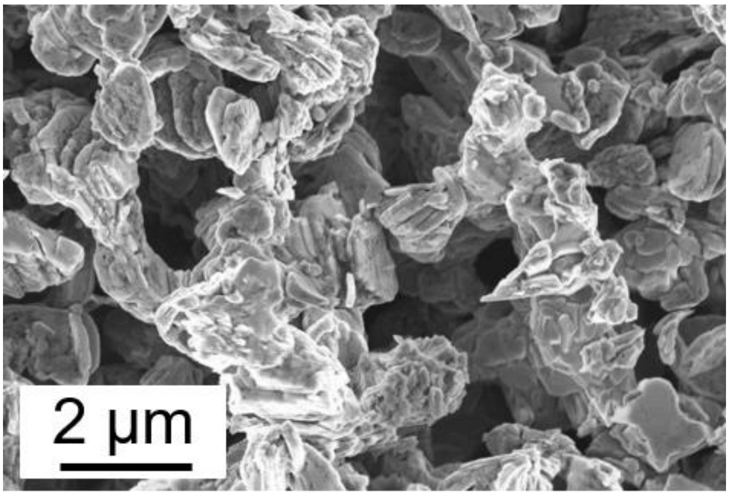

Figure S3: Exemplary SEM image of primary KVP/C particles synthesized with 5 wt.% sucrose at 750°C under Ar + 3 % H_2 atmosphere.

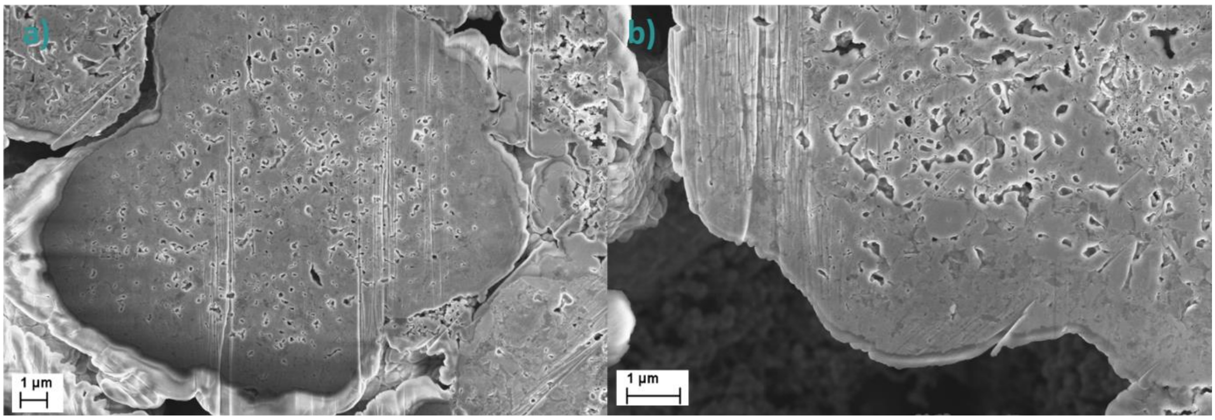

Figure S4: SEM images of KVP/C-3 to visualize the dense surface of the KVP/C-3 granules.

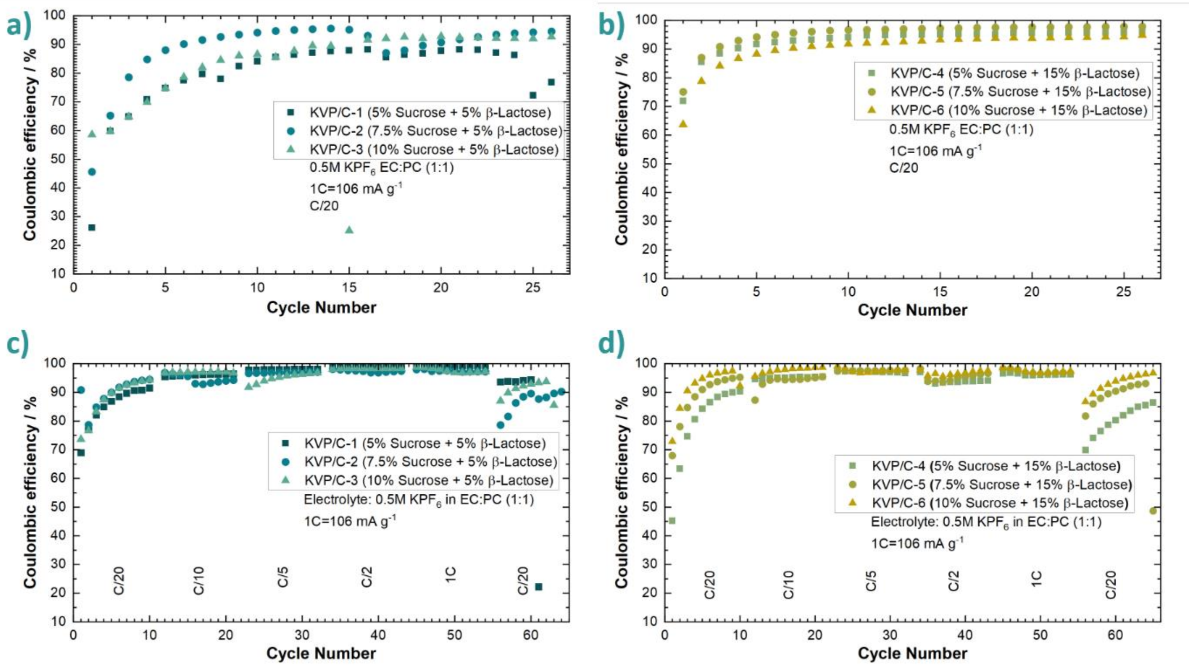

Figure S5: Coulombic Efficiencies of the KVP/C samples tested in potassium ion half cells at C/20 a) & b) and at different C-rates c) & d).

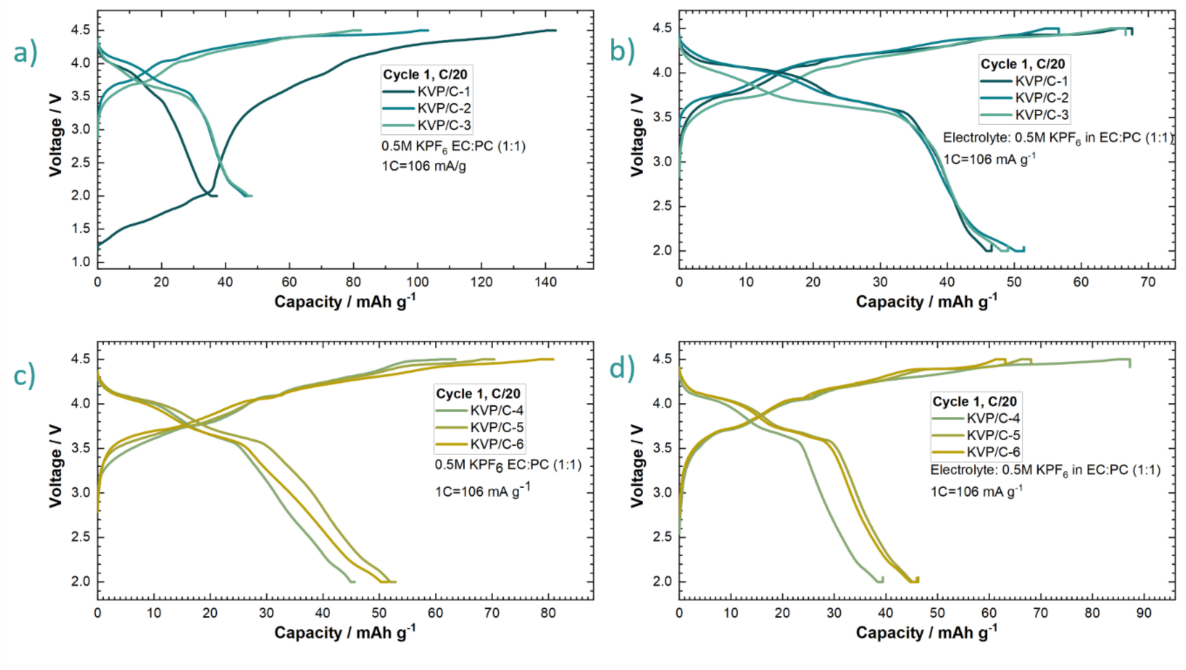

Figure S6: Voltage profiles for the 1st cycles of a) KVP/C samples with 5% β-lactose during C/20, b) KVP/C samples with 5% β-lactose during C-Rate test, c) KVP/C samples with 15% β-lactose during C/20, d) KVP/C samples with 15% β-lactose during C-Rate test.

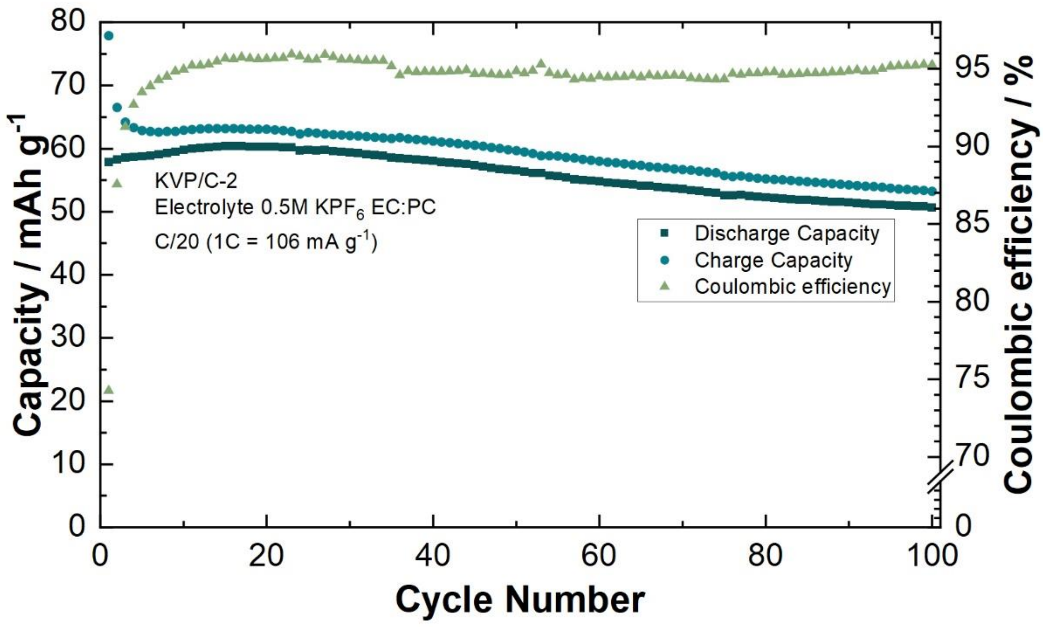

Figure S7: Cycle Life evaluation of KVP/C-2 at C/20 for 100 cycles in potassium ion half cells with the corresponding charge/discharge capacities and Coulombic efficiency.

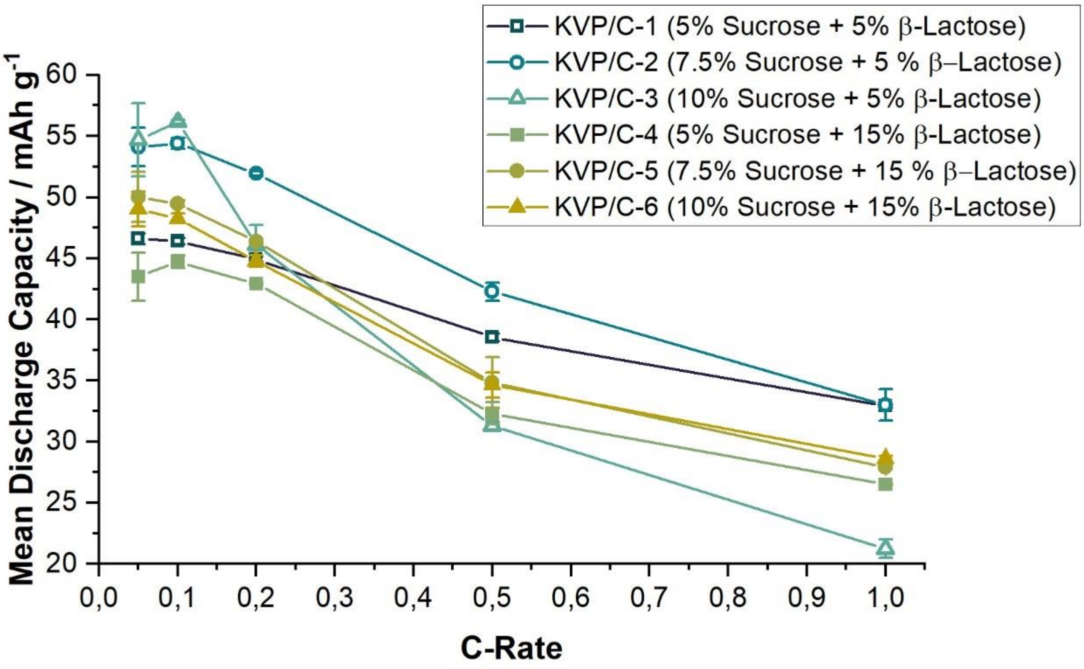

Figure S8: Mean Discharge Capacity over C-rate for all different KVP/C-composites.

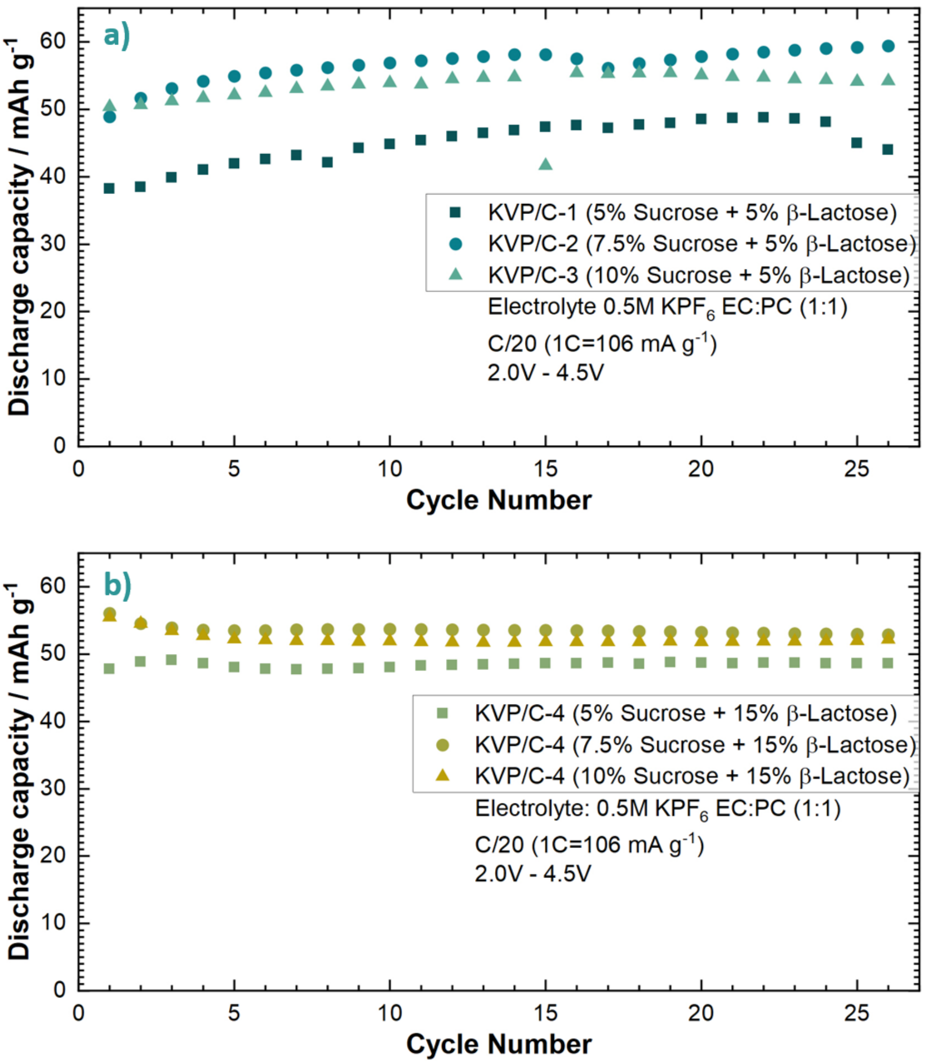

Figure S9: Discharge Capacity of KVP/C samples at C/20 in PIB half cells without carbon content (see Table 3): a) KVP/C samples with 5 wt.% β-lactose and b) KVP/C samples with 15 wt.% β-lactose.

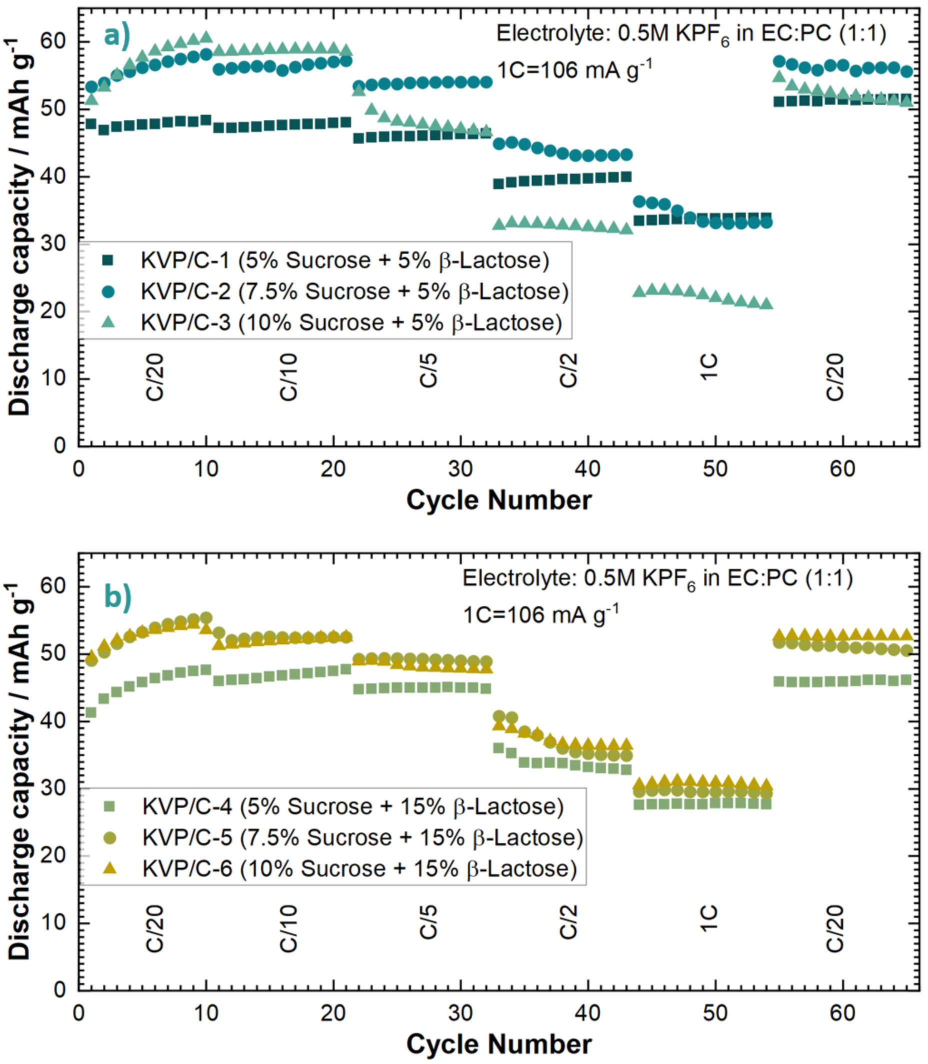

Figure S10: C-Rate capability of KVP/C samples in PIB half cells without carbon content (see Table 3): a) KVP/C samples with 5 wt.% β-lactose and b) KVP/C samples with 15 wt.% β-lactose.

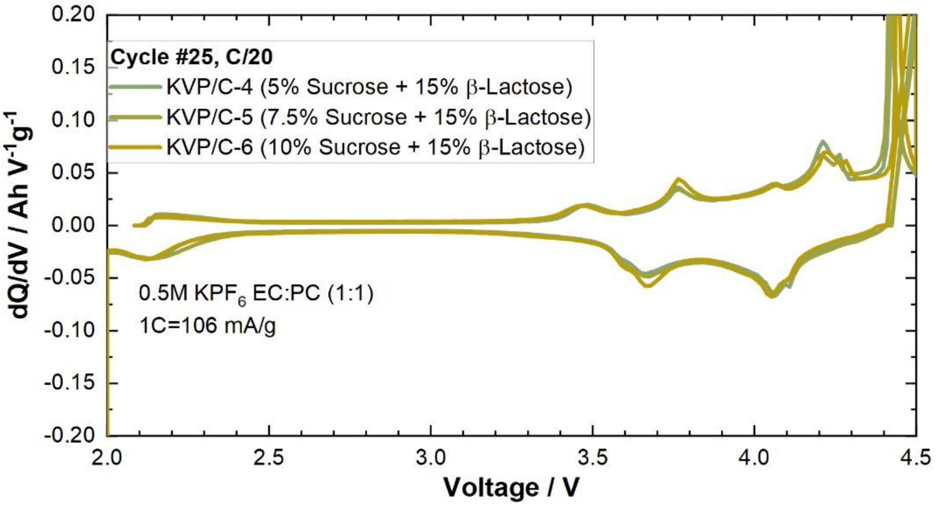

Figure S11: dQdV-Plot of KVP/C samples with 15 wt.% β-lactose in PIB half cells at C/20 in the 25th cycle.

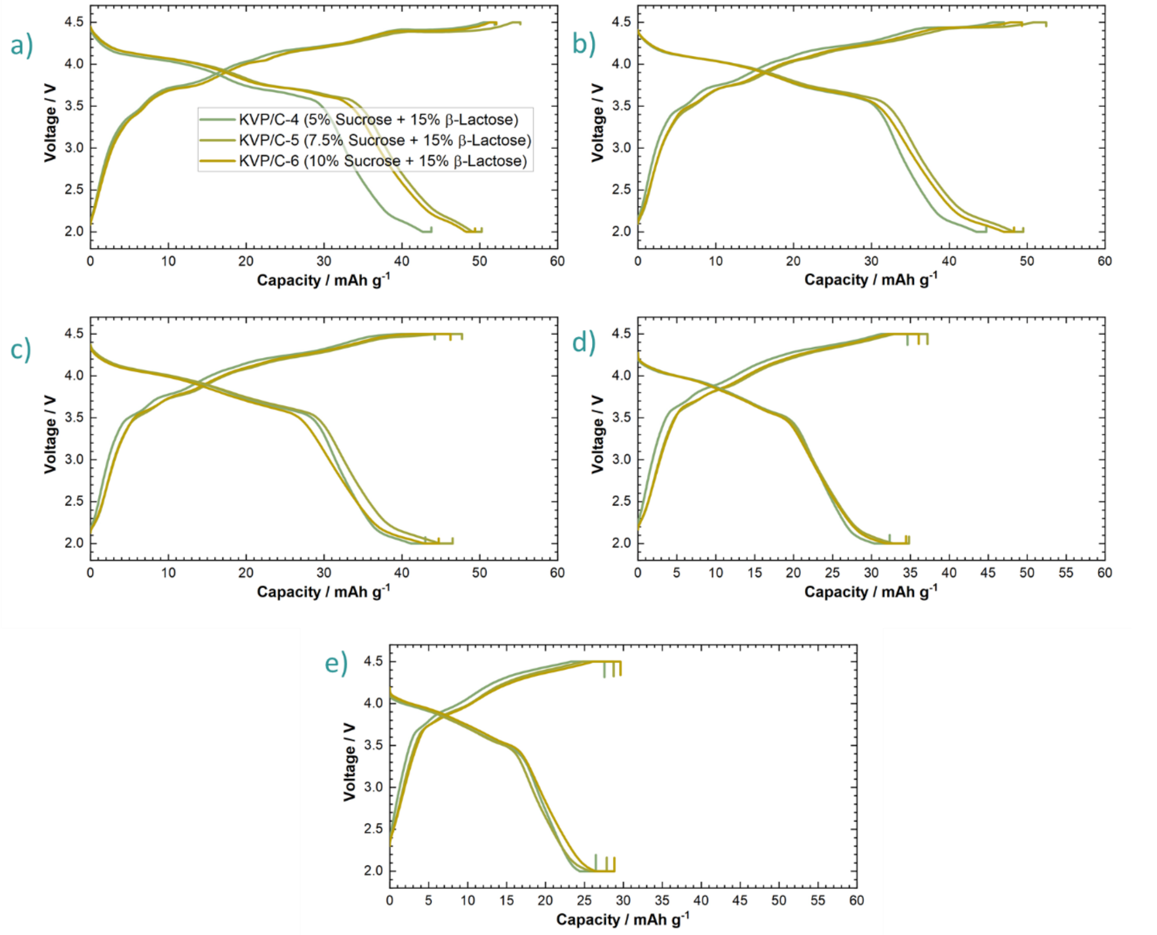

Figure S12: Voltage Profiles of KVP/C samples with 15 wt.% of β-lactose in PIB half cells at different C-Rates in the 5th cycle at each C-rate. a) C/20, b) C/10, c) C/5, d) C/2 and e) 1C. 1C=106 mA g^{-1} and as an electrolyte 0.5M KPF_6 in EC:PC (1:1) was used.

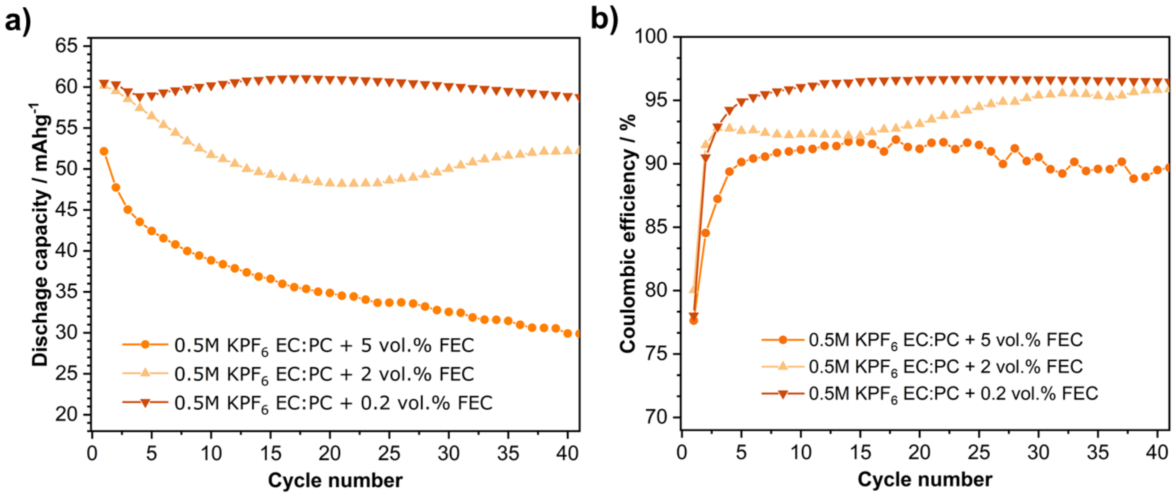

*Figure S13: **a)** Discharge capacities and **b)** Coulombic efficiency of FEC containing electrolytes in multiple concentrations.*

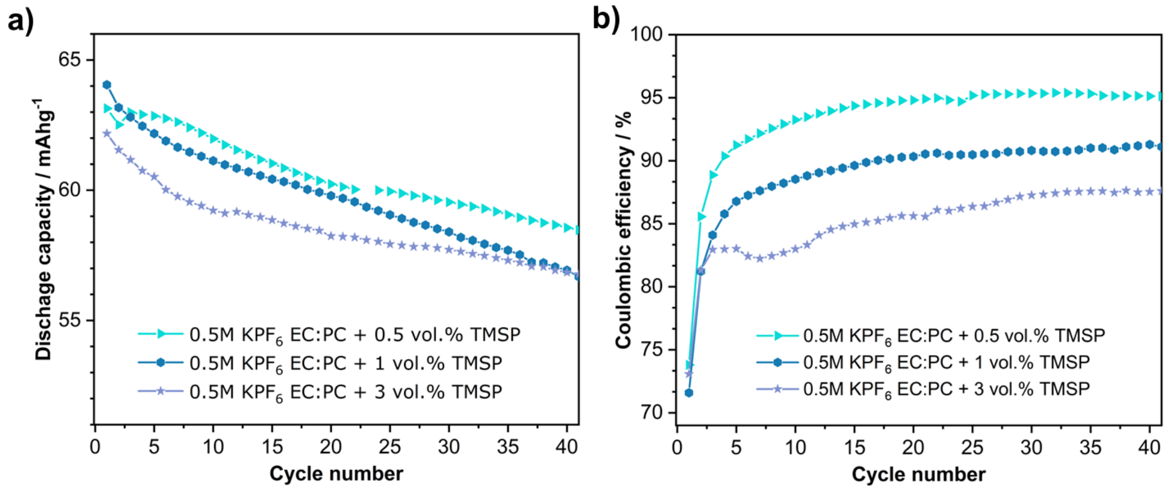

*Figure S14: **a)** Discharge capacities and **b)** Coulombic efficiency of TMSP containing electrolytes in multiple concentrations.*

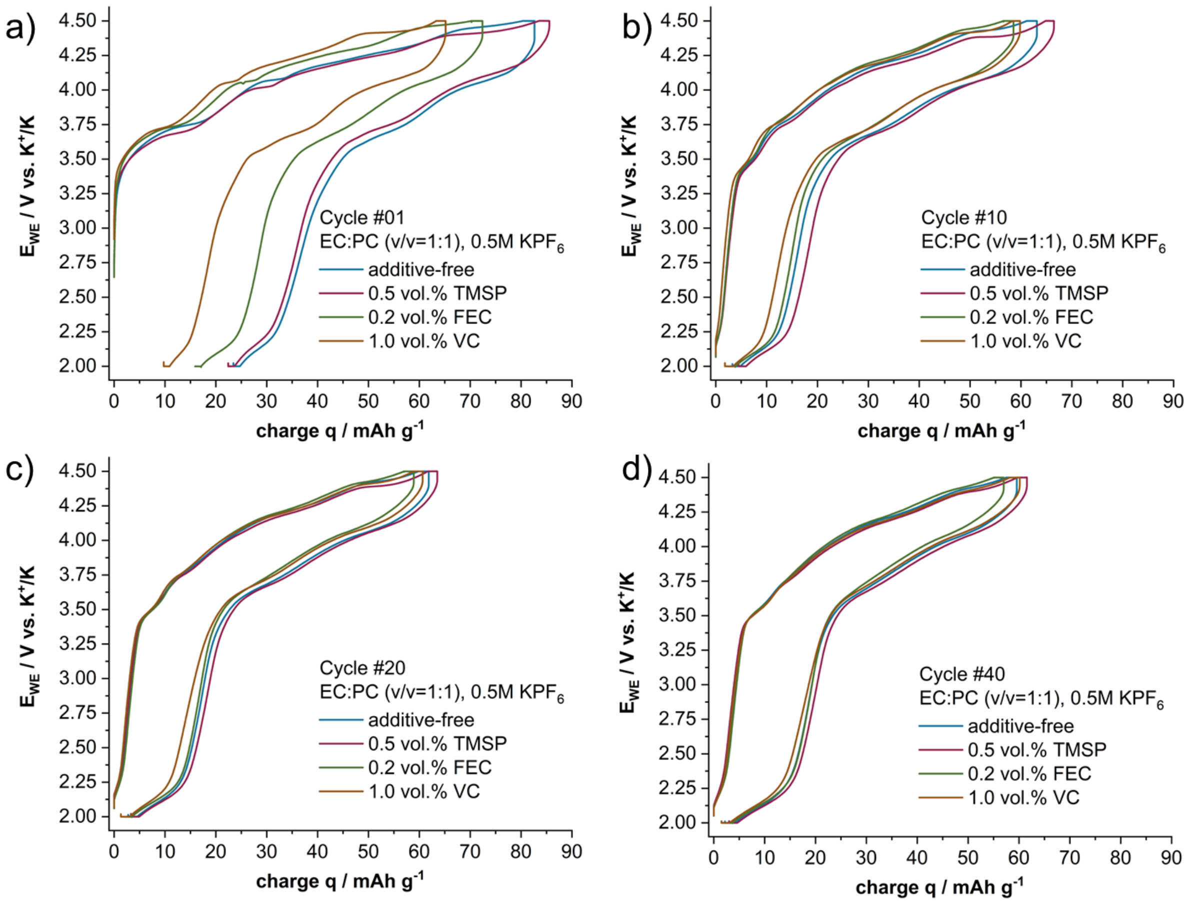

Figure S15: Voltage profiles of KVP/C electrodes tested in two-electrode half cells in EC:PC (v/v=1:1) based electrolytes with 0.5 M KPF_6 with either no additive, 0.5 vol.% TMSP, 0.2 vol.% FEC or 1.0 vol.% VC on the first cycle (a), 10th cycle (b), 20th cycle (c) and 40th cycle (d).

Table S1: Theoretical and experimental carbon content of the KVP/C-samples after calcination.

Sample	Sucrose Content [wt.%]	Theoretical Carbon Content [wt.%]	Experimental Carbon Content [wt.%]	Carbon used for Reduction of Vanadium [wt.%]
KVP/C-1/4	5	2.1	0.9	1.2
KVP/C-2/5	7.5	3.2	2.3	0.9
KVP/C-3/6	10	4.2	3.8	0.4

Table S2: Specific surface after calcination at 750°C depending on the sucrose content.

Sample	Sucrose-Content [wt.%]	Specific Surface [m^2/g]
KVP/C	5	3.3
	7.5	9.5
	10	27.7

Table S3: K:V:P ratio of sample KVP/C-2 determined by an ICP-OES analysis.

Sample	K	V	P
KVP/C-2	3.0	2.03	3.08
